# Association between human papillomavirus vaccine status and other cervical cancer risk factors

**DOI:** 10.1016/j.vaccine.2014.06.011

**Published:** 2014-07-23

**Authors:** Harriet L. Bowyer, Rachael H. Dodd, Laura A.V. Marlow, Jo Waller

**Affiliations:** Health Behaviour Research Centre, Department of Epidemiology & Public Health, UCL, Gower Street, London WC1E 6BT, United Kingdom

**Keywords:** HPV, Vaccination, Cervical cancer, Cancer risk, Adolescent, Immunisation

## Abstract

Little is known about the relationship between HPV vaccine uptake and other risk factors for cervical cancer. This study aimed to measure the association between vaccine status and cervical cancer risk factors in adolescent girls. Girls (15–16 years) from the first two cohorts to be offered routine HPV vaccination in the NHS immunisation programme completed a survey 3 years post-vaccination. Recruitment took place at 13 schools in London. Of 2768 girls registered in Year 11, 1912 (69%) took part and provided analysable data. Questions assessed vaccine status, demographic characteristics, smoking status, sexual behaviour and intention to attend cervical screening. Overall, 78% had completed the three-dose vaccine course. There was no association between vaccine status and smoking behaviour or sexual experience. In adjusted analyses, girls from black or ‘other’ ethnic backgrounds were less likely to be fully-vaccinated than those from white backgrounds. Those with low intentions to attend cervical screening were less likely to be fully vaccinated than those with high intentions. Efforts will be needed to ensure that unvaccinated women understand the importance of cervical screening when they reach the age that screening begins. Ethnic inequalities in vaccine coverage need to be explored further.

## Highlights

• Girls from black or ‘other’ ethnic backgrounds were less likely to be vaccinated than white girls.
• Vaccine status was not associated with smoking status or sexual activity.
• Unvaccinated girls had lower intentions to attend cervical screening in the future.

## 1 Introduction

In England, girls age 12–13 years are offered free human papillomavirus (HPV) vaccination in a school-based programme launched in 2008. The programme has achieved high coverage, with latest figures showing that 84% and 81% of eligible girls in the first (2008/9) and second (2009/10) cohorts to be offered the HPV vaccine have received all three doses as recommended [1]. This relatively new cervical cancer control policy is complemented by a long-standing call–recall screening programme for women aged 25–64 years, in which women receive regular screening invitations by post. Women aged 25–49 years are invited every 3 years and women aged 50–64 years are invited every 5 years. Written invitations ask women to make an appointment for a Pap test with their general practitioner or primary care nurse. The programme is funded by the NHS and is free at the point of delivery. Screening uptake in women aged 25–64 years is high, with 78% having been screened at least once in the previous 5 years [2].

Despite the successful screening programme, almost 3000 women are diagnosed with cervical cancer each year in the UK, and about 900 women die of the disease [3]. Modelling studies have estimated that 80% vaccine coverage will result in a 63% decrease in cervical cancer incidence in 20–29 year old women by 2025 [4]. However this assumes an equal level of baseline risk of cervical cancer in vaccinated and unvaccinated girls. If unvaccinated girls are, in fact, at higher risk of cervical cancer for reasons other than their vaccination status (e.g. early sexual debut, smoking or non-attendance at screening), then the true impact of the vaccination programme may be less than has been anticipated. In their modelling study, Cuzick and colleagues acknowledge that it is unknown whether non-participation in vaccination and screening will be independent of one another. They raise the possibility that vaccinated women may perceive less need for screening, but also that factors like deprivation may be associated with non-participation in both programmes [4]. The relationship between vaccine status and screening participation in England will not be apparent until about 2021, when the cohort vaccinated in 2008 will be eligible for screening. The full impact of the vaccine on cervical abnormalities and cancer will not be seen until even later.

Currently, the major determinant of cervical cancer risk in England is screening attendance [5]. Screening attendance is demographically patterned, with non-white women and those with less education and from lower socioeconomic status (SES) backgrounds being less likely ever to attend screening [6–9]. Other major risk factors for cervical cancer are having many sexual partners, due to an increased risk of HPV acquisition [10], and cigarette smoking [11–13]. Smoking status is strongly related to SES [14] and ethnicity [15]; and sexual behaviour also varies by ethnic group [16]. Associations between sexual behaviour and SES are less clear-cut [17] but women with academic qualifications and managerial/professional occupations are at lower odds of having intercourse before the age of 16 [18].

There is emerging evidence that these risk factors for cervical cancer may also be related to HPV vaccination status. Non-white women are less likely to have been vaccinated than white women in the UK and elsewhere [19,20], and black ethnic groups are particularly unlikely to be vaccinated in the US [21]. The role of religion in vaccine initiation is less clear [21]. A social gradient in HPV vaccination uptake has been observed in the UK catch-up cohorts [22], but is less clear in the routine cohorts [23–25].

In most cases HPV vaccination is offered some years before cervical screening and therefore few studies have examined the association between uptake of HPV vaccination and cervical screening attendance. Studies in Australia [26] and Germany [27] that have explored this have found no significant association, but samples have been small and have tended to include older women who received the vaccine on an opportunistic basis. A larger study conducted as part of an evaluation of the immunisation programme in Scotland found higher intentions to attend future cervical screening in vaccinated girls [28], and a study in Wales found that unvaccinated women from the catch-up cohort were less likely to attend screening when invited at age 20 [29]; however no such research has yet been conducted in England.

This study aimed to establish whether unvaccinated girls are likely to be at disproportionately higher risk of cervical cancer. We used data collected from vaccinated and unvaccinated girls in the first two cohorts of the HPV immunisation programme to consider the association between vaccine status and (i) demographic risk factors and (ii) behavioural risk factors for cervical cancer.

## 2 Materials and methods

### 2.1 Design and participants

Assuming that vaccine coverage (three doses) would be 77.5% [30], we determined that a sample size of 2000 would include approximately 450 unvaccinated girls, giving us 80% power to detect a 6% difference in the proportion of these girls who would be sexually active by the time of the survey, compared with the vaccinated girls (alpha = .05). We therefore set a target of recruiting 2000 participants over two cohorts.

Female adolescents in UK school Year 11 (age 15–16 years) were recruited from 13 state-funded schools across London, England in September 2011. In 2008/9 these girls were in the first cohort to be offered the bivalent HPV vaccine at school in Year 8. A sampling frame was used to randomly select state-funded schools that varied in terms of SES and HPV vaccine uptake. Only schools that achieved vaccine uptake levels within ±10% of the national average in 2008/9 (80%) [30] were included (*n* = 89), to eliminate schools where uptake might be unusually high or low for idiosyncratic reasons related to delivery rather than the individual characteristics that were the focus of this study. Schools were classified as having achieved uptake rates above or below the national average. School-level SES was measured using General Certificate in Secondary Education (GCSE) attainment and Free School Meal Eligibility (children are eligible for free school meals if their parents are entitled to means-tested welfare benefits from the UK government [31]). Schools were classified as being above or below the national average on each of these measures [32,33]. Schools were randomly selected from each cell of the sampling frame and contacted via email and telephone until we reached an estimated target sample of 1000 participants, based on school roll numbers. Further details about the sampling frame have been reported elsewhere [34].

All 89 schools were sent details of the study; 13 schools agreed to participate, 19 refused due to scheduling difficulties and 57 did not respond to our initial contact and were not re-contacted because the target sample had been achieved. One year later, in September 2012, female adolescents in school Year 11 were recruited from 12 of the original 13 schools; one school withdrew from the study because of scheduling difficulties. These girls were in the second cohort offered the routine HPV vaccine at school (in 2009/10). Identical materials and methods were used during the two waves of data collection.

Parents received an information sheet about the study and an opt-out form 1 week before the research took place. Parental consent was implied if the opt-out form was not returned to the school. All girls in attendance were given an information sheet and a questionnaire booklet. Consent was implied upon completion of the questionnaire and all girls were debriefed with an information sheet containing information about HPV. The study was approved by UCL research ethics committee (ref: 0630/002).

### 2.2 Measures

#### 2.2.1 Demographic characteristics

Participants were asked to report their age, ethnicity, religion and, if they reported a religious affiliation, to say whether they practised their religion. Household wealth was measured using the Family Affluence Scale [35]; a validated self-report measure for adolescents. This measure asks adolescents how many vehicles and computers their family owns, whether they have a bedroom to themselves and how many holidays they have had with their family in the past year. Items were summed to give an overall family affluence score (range 0–10), which was split into tertiles: ‘low’ (scores of 0–4), ‘medium’ (scores of 5–6) and ‘high’ (scores of 7–10).

#### 2.2.2 Risk factors for cervical cancer

Participants were asked whether they smoked (yes/no). Sexual experience was assessed by asking participants ‘Have you ever had vaginal sex?’ (yes/no); this question was adapted from the ‘National Survey of Sexual Attitudes and Lifestyles’ [17]. Expectation of having sex in the next year was also assessed using two items adapted from Sheeran and Orbell [36]: ‘I expect I will have sex this year’ and ‘I think I will have sex this year’ (5-point scale: ‘strongly disagree’ to ‘strongly agree’, scored from 1 to 5). These items correlated highly (*r* = 0.97) and were summed to give an overall score which was split into tertiles: ‘no expectation’ (scores of 2), ‘low expectation’ (3–5) and ‘high expectation’ (6–10) of having sex in the next year. Intention to attend cervical screening in the future was assessed using similar items: ‘When I am older and am invited to go for a smear (Pap) test, I intend to go’ and ‘When I am older and am invited to go for a smear (Pap) test, I will try to go’ (with a 5-point response scale as before). The items correlated highly (*r* = 0.89) and were summed to give an overall screening intention score which was split into tertiles: ‘low intention’ (scores of 2–6), ‘medium intention’ (7–8) and ‘high intention’ (9–10). Other measures in the questionnaire that are not reported here have been described elsewhere [34].

#### 2.2.3 Vaccine status

After reading a brief description of the HPV vaccine (see Box 1) participants were asked to indicate their vaccine status (response options: ‘I have had all 3 doses of the HPV vaccine’; ‘I have had 1 or 2 doses of the HPV vaccine’; ‘I have been offered the HPV vaccine but I haven’t had it’; ‘I have not been offered the HPV vaccine’; ‘I don’t know’).

### 2.3 Analysis

Logistic regression analyses, clustering by school and cohort, were used to examine the association between HPV vaccine status (fully vaccinated versus un-/under-vaccinated) and other risk factors for cervical cancer. It is necessary to adjust for clustering of data within schools and cohorts in order to obtain unbiased tests of significance. Analyses were performed using the Complex Samples function in SPSS v.20 [37].

## 3 Results

### 3.1 Sample characteristics

**Fig. 1:**
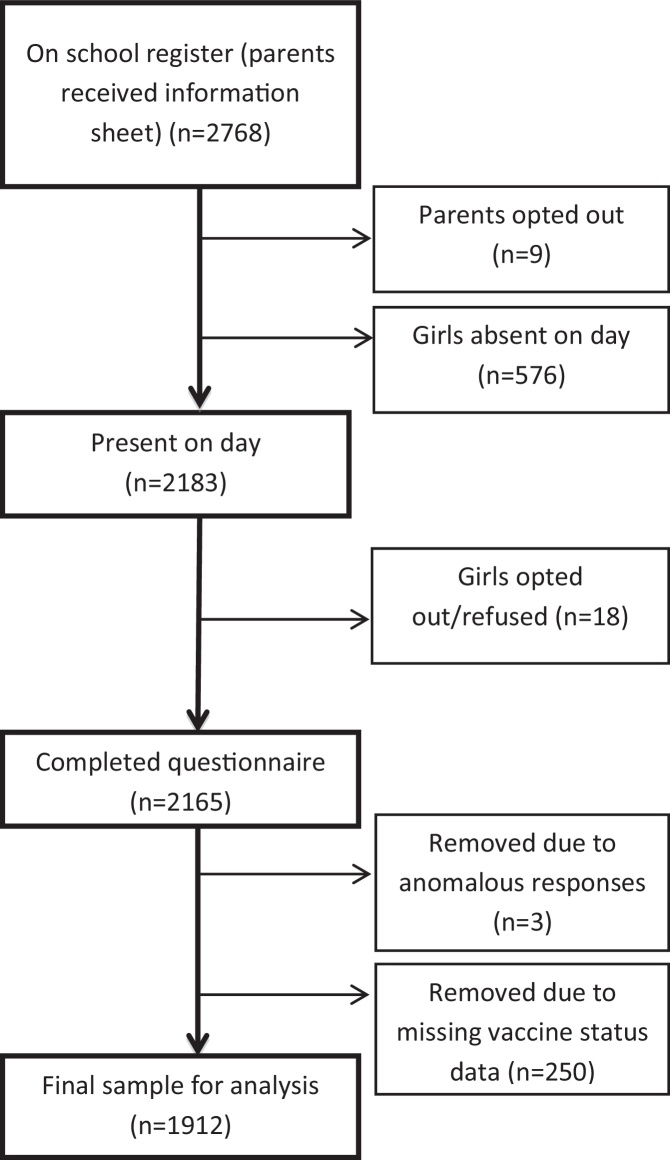
Recruitment and sample size for data analysis.

A total of 2162 girls agreed to participate in the study (*n* = 1033 from the 2008/9 cohort and *n* = 1129 from the 2009/10 cohort) (see Fig. 1). The overall participation rate among girls in attendance at the point of data collection was over 98% across both years. Eighteen girls and nine parents refused consent and based on the school role numbers provided 576 were absent at the time of data collection. In some cases, girls may have been present at school but missed the data collection session due to other commitments. Other reasons for absence are unknown. Respondents who did not know their HPV vaccination status (*n* = 221/2162; 10.2%) or who failed to report their vaccine status (*n* = 29/2162; 1.3%) were excluded from analyses, leaving a sample of 1912 (69.1% (1912/2768) of the total eligible population. Individuals who reported having received all three doses of the HPV vaccine were coded as ‘fully vaccinated’ (*n* = 1499/1912; 78.4%). Participants who reported receiving one or two doses of the HPV vaccine (*n* = 122/1912; 6.4%), had been offered the vaccine but had not had it (*n* = 233/1912; 12.2%) or had not been offered the vaccine (*n* = 58/1912; 3.0%) were coded as ‘un/under-vaccinated’ (*n* = 413/1912; 21.6%). Vaccine status was coded in this way because it seemed unlikely that three years on, under-vaccinated girls would receive any additional doses of the vaccine and these girls may therefore be at higher risk of cervical cancer.

Demographic characteristics of the sample are shown in Table 1. The sample was ethnically diverse with only 44.2% reporting being from a white background (*n* = 845/1912). The largest religious group was Christian (*n* = 814/1912; 42.6%) and overall 40.1% of respondents reported practising a religion (*n* = 767/1912). The mean Family Affluence Score was 5.57 (SD = 1.92; range: 0–10). There were some significant differences between cohorts (see Table 1 for *p*-values). More girls in the first cohort were Christian (45% vs. 40%) while more in the second cohort had no religion (33% vs. 27%). Girls in the first cohort were more likely to report having had vaginal sex (20% vs. 16%) and had higher screening intentions than girls in the second cohort (35% vs. 28%).

### 3.2 Vaccine status and demographic risk factors

In unadjusted analyses there was a significant association between vaccine status and ethnicity; girls from all non-white ethnic backgrounds were significantly less likely to be fully vaccinated than those from white ethnic backgrounds (white: 85%, non-white: 69–78%; see Table 2). There was also a significant association between vaccine status and religion; girls with no religious affiliation were more likely to be fully vaccinated than Christian girls (85% vs. 77%). There appeared to be a linear association between vaccine status and family affluence, but this did not reach statistical significance. There was no association between vaccine status and religiosity. After adjusting for ethnicity, religion was no longer significantly associated with vaccine status. However, the ethnicity association remained significant after adjusting for religion; girls from black and ‘other’ ethnic backgrounds were less likely to be fully vaccinated than girls from white backgrounds.

### 3.3 Vaccine status and behavioural risk factors

There was no association between vaccine status and current risk behaviours: smoking status or sexual experience. There was no association between vaccine status and expectation of having sex in the next year; however cervical screening intentions were associated with vaccine status. Those with low intentions to attend cervical screening in the future were significantly less likely to be fully vaccinated compared with those who had high intentions (70% vs. 81%). This association remained significant after adjusting for ethnicity and religion.

## 4 Discussion

This study showed that compared with fully vaccinated girls, those who had not received all three doses were more likely to be from non-white ethnic backgrounds and to have lower intentions to attend for cervical screening in the future. These results support previous studies that suggest non-white ethnicity is associated with being un/under-vaccinated [19–21] and that unvaccinated girls may be less likely to attend cervical screening [28,29]. Encouragingly, we found no evidence of an association between vaccination status and socioeconomic status, sexual behaviour or cigarette smoking; again, supporting previous findings that vaccination status does not influence sexual behaviour [38,39] and that coverage is not associated with area-level deprivation [25]. It is likely that the association between vaccination uptake and participation in screening is explained by a general interest in health among those who engage in health protective behaviours. Alternatively, some studies suggest that women who attend cervical screening are more likely to vaccinate their daughters against HPV [40–43], so it is possible that the screening intentions expressed by the vaccinated girls in our sample were reflective of their mothers’ behaviour. We did not measure parental screening behaviour, but future studies should consider this possibility. Exposure to information about cervical screening during the HPV vaccination campaign (through leaflets, providers or discussions with their parents) could also explain increased intention to attend for screening in vaccinated girls, although all girls offered the vaccine are exposed to written information on screening, regardless of uptake. In additional analyses (not reported here) the association between vaccination status and intention to be screened remained significant after adjusting for previous awareness of cervical cancer screening, suggesting that attitudes rather than knowledge underpin this association.

The association between vaccination status and screening intention is concerning because it suggests there will be a distinct group of women who remain unvaccinated and unscreened, and will therefore be at increased risk of cervical cancer. If this is the case, the expected impact of the vaccination programme on cervical cancer mortality, as estimated in modelling studies [4], may be reduced. It will therefore be critically important to highlight the need for screening, particularly for unvaccinated women, in materials sent with future screening invitations to these cohorts. Of course, this study measured screening intention almost 10 years before girls were due to be invited, and it is unclear to what extent this will reflect their future behaviour.

The findings relating to ethnicity are also concerning, particularly as fewer women from non-white ethnic backgrounds tend to be screened for cervical cancer in the UK and elsewhere [6,44]. Rates of cervical cancer in women from black and Asian backgrounds have been found to be higher than for white women in the 65+ age-group [45]. Incidence in women under 65 is currently lower among Asian women but is similar among black and white women, so lower vaccine uptake in black girls is of particular concern. Uptake may be low in non-white ethnic groups due to cultural barriers and parental concerns that vaccination may encourage sexual activity [46]. Studies have suggested the role of social sources of information and discussion (e.g. hearing about the HPV vaccine and discussing it with family or friends) are important for increasing perceived vaccine effectiveness [47] and increasing requests for the vaccine [48]. This supports previous research showing cues to action (e.g. a recommendation from friends, family or a doctor) are the strongest predictors of vaccine uptake [49]. These factors should be taken into consideration when developing health promotion campaigns (e.g. narrative leaflets) aimed at reducing ethnic inequalities in vaccine uptake.

As increasing numbers of countries, including the UK, move to a two-dose HPV vaccine schedule [50], ethnic inequalities might be reduced. Research in the US has shown that ethnic disparities occur mainly between initiators and completers, with those from non-white ethnic backgrounds being equally likely to initiate but less likely to complete the three dose course [51]. As we had a single response category for ‘1–2’ doses, we were unfortunately unable to explore predictors of receipt of two or more doses in our sample.

This study benefited from a large sample size, including girls from a variety of ethnic and socioeconomic backgrounds. Response rates in both waves of data collection were very high at over 98% but we acknowledge that there could be systematic differences between the schools that readily agreed to take part in the study and those that refused or failed to respond to our initial contact. In addition, a significant number of girls were absent at the point of data collection or did not know their vaccine status, which may reduce the generalisability of the findings. Because recruitment was limited to London, and to schools with levels of vaccine coverage within 10% of the national average, the results may not be generalisable to England more widely or to schools where uptake is much higher or lower.

Self-reported uptake of the three-dose vaccine among girls in our sample (80% and 77% in the first and second cohorts respectively) was similar to figures for national uptake (84% in 2008/9; 81% in 2009/10) [1]. Our findings are likely to be more generalisable than those of previous studies in cohorts offered the HPV vaccine opportunistically [26,27]. Vaccination status was self-reported which may have limited reliability 3 years post-vaccination. Around 10% of respondents did not know their vaccine status, and there was some variation between reported levels of vaccination in our sample and levels recorded by the Primary Care Trusts in which the schools were located (data not reported). We were unable to validate individual-level vaccine status due to the need to assure anonymity. As estimates of the accuracy of self-reported HPV vaccine status vary, more research in this context is warranted [52,53].

The 10% of girls who responded ‘don’t know’ to the vaccine status question were similar in terms of demographic and behavioural risk factors to girls who were un/under-vaccinated (analyses not reported). We repeated our regression analyses including these girls in the un/under-vaccinated group, and found very similar results to those reported here, suggesting that these girls were unlikely to be fully vaccinated.

## 5 Conclusion

Our results suggest that un/under-vaccinated girls in England may be at disproportionately greater risk of cervical cancer due not only to their vaccine status, but also their low screening intentions. Efforts will be needed to ensure that un/under-vaccinated women understand the importance of cervical screening when they reach the age that screening invitations begin. There is also an urgent need to understand ethnic inequalities in vaccination uptake.

## Conflict of interests

All authors declare no conflict of interest that may have influenced this work.

## Contributions

JW conceptualised and designed the study. HB and JW collected and analysed the data for the study and all authors contributed to the interpretation and the writing of this paper and have approved the final draft.

## Funding

This study was funded as part of a larger project grant from Cancer Research UK (Grant reference A13254).

## Figures and Tables

**Table 1:** Descriptive characteristics of the total sample (*N* = 1912).

	Total (*N* = 1912^a^)	Column% (*n*)		
		2008/9 cohort (*n* = 918^a^)	2009/10 cohort (*n* = 994^a^)	*X*^2^ (*p*-value)
Vaccine status				.070
Fully vaccinated	78.4 (1499)	80.2 (736)	76.8 (763)	
Un/under-vaccinated	21.6 (413)	19.8 (182)	23.2 (231)	
Ethnicity				.327
White	44.2 (845)	44.9 (412)	43.6 (433)	
Asian	19.2 (367)	19.1 (175)	19.3 (192)	
Black	22.0 (421)	23.0 (211)	21.1 (210)	
Other	13.7 (262)	12.3 (113)	15.0 (149)	
Religion				.035
Christian	42.6 (814)	45.4 (417)	39.9 (397)	
None	30.0 (573)	27.0 (248)	32.7 (325)	
Muslim	18.7 (357)	18.7 (172)	18.6 (185)	
Other	8.2 (157)	8.4 (77)	8.0 (80)	
Religiosity				.980
Practising	40.1 (767)	41.6 (382)	38.7 (385)	
Not practising	29.4 (563)	30.5 (280)	28.5 (283)	
Family Affluence Scale				.156
Low affluence (0–4)	29.1 (556)	28.4 (257)	30.5 (299)	
Medium affluence (5–6)	37.2 (711)	40.0 (362)	35.6 (349)	
High affluence (7–10)	32.3 (618)	31.7 (287)	33.8 (331)	
Smoking status				.159
No	85.4 (1632)	84.3 (774)	86.3 (858)	
Yes	12.8 (245)	13.9 (128)	11.8 (117)	
Sexual experience (vaginal sex)				.031
No	78.5 (1500)	80.4 (716)	84.2 (784)	
Yes	16.8 (322)	19.6 (175)	15.8 (147)	
Cervical screening intention				.002
Low (2–6)	22.9 (437)	23.7 (189)	29.8 (248)	
Medium (7–8)	36.0 (688)	41.7 (333)	42.6 (355)	
High (9–10)	26.5 (506)	34.6 (276)	27.6 (230)	
Expectation of having sex in the next year				.226
No expectation (2)	36.2 (692)	37.9 (329)	39.6 (363)	
Low expectation (3–5)	19.5 (373)	19.8 (172)	21.9 (201)	
High expectation (6–10)	37.7 (721)	42.3 (368)	38.5 (353)	

a *n* varies because of missing data.

**Table 2:** Demographic and lifestyle predictors of being fully vaccinated against HPV, clustering by school and cohort (*n* = 1912^a^).

	Row% Fully vaccinated (*n* = 1499^a^)	Row% Un/under-vaccinated (*n* = 413^a^)	Unadjusted analyses		Adjusted analyses (*n* = 1608)	
			OR (95% CI)	*p*-Value	OR (95% CI)	*p*-Value
Demographic risk factors						
Ethnicity						
White	85.4 (722)	14.6 (123)	1.00		1.00	
Asian	77.7 (285)	22.3 (82)	**0.59 (0.38–0.93)**	**.023**	0.67 (0.38–1.19)	.162
Black	68.6 (289)	31.4 (132)	**0.37 (0.24–0.59)**	**<.0001**	**0.41 (0.27–0.60)**	**<.0001**
Other	74.0 (194)	26.0 (68)	**0.49 (0.34–0.69)**	**<.0001**	**0.56 (0.38–0.82)**	**.005**
Religion						
Christian	76.7 (624)	23.3 (190)	1.00		1.00	
None	85.3 (489)	14.7 (84)	**1.77 (1.16–2.70)**	**.010**	1.28 (0.88–1.86)	.453
Muslim	70.3 (251)	29.7 (106)	0.72 (0.51–1.02)	.065	0.77 (0.52–1.15)	.186
Other	80.9 (127)	19.1 (30)	1.29 (0.69–2.41)	.410	1.28 (0.66–2.51)	.194
Religiosity						
Practising	73.0 (560)	27.0 (207)	1.00			
Not practising	79.0 (445)	21.0 (118)	1.39 (0.99–1.96)	.054		
Family Affluence Scale						
High affluence (7–10)	81.9 (506)	18.1 (112)	1.00			
Medium affluence (5–6)	77.9 (544)	22.1 (157)	0.78 (0.61–1.01)	.056		
Low affluence (0–4)	76.1 (423)	23.9 (133)	0.70 (0.47–1.06)	.091		
Behavioural risk factors						
Smoking status						
No	78.4 (1280)	21.6 (352)	1.00			
Yes	78.8 (193)	21.2 (52)	0.98 (0.65–1.47)	.918		
Sexual experience (vaginal sex)						
No	78.9 (1184)	21.1 (316)	1.00			
Yes	78.0 (251)	22.0 (71)	0.94 (0.68–1.31)	.717		
Cervical screening intention						
High intention (9–10)	81.4 (412)	18.6 (94)	1.00		1.00	
Medium intention (7–8)	77.9 (536)	22.1 (152)	0.81 (0.55–1.18)	.248	0.87 (0.60–1.27)	.467
Low intention (2–6)	70.3 (307)	29.7 (130)	**0.54 (0.39–0.75)**	**.001**	**0.63 (0.45–0.89)**	.010
Expectation of having sex in the next year						
High expectation (6–10)	81.7 (589)	18.3 (132)	1.00			
Low expectation (3–5)	80.7 (301)	19.3 (72)	1.07 (0.74–1.55)	.721		
No expectation (2)	76.2 (527)	23.8 (165)	1.40 (0.90–2.16)	.127		

a *n* may vary because of missing data, bold text indicates significance at *p* < .05.

## Box 1

Human papillomavirus (HPV) is a very common infection involved in most cervical cancer.

It is transmitted via skin-to-skin contact, most commonly during sexual activity.

A vaccine was developed that protects against this infection.

You should have been offered the HPV (cervical cancer) vaccine in Year 8.

It involved having three injections over about 6 months.
